# Polysomnographic correlates of self-and caregiver-reported sleep problems in post-stroke patients

**DOI:** 10.3389/fneur.2025.1587378

**Published:** 2025-07-16

**Authors:** Kamalesh Tayade, Deepti Vibha, Rajesh Kumar Singh, Awadh Kishor Pandit, Bhargavi Ramanujam, Animesh Das, Arunmozhimaran Elavarasi, Ayush Agarwal, Achal Kumar Srivastava, Manjari Tripathi

**Affiliations:** Department of Neurology, All India Institute of Medical Sciences, New Delhi, India

**Keywords:** post-stroke sleep disturbances, OSA (obstructive sleep apnea), SDB (sleep-disordered breathing), periodic leg movement disorder, polysomnography, STOP-BANG

## Abstract

**Background:**

Post-stroke sleep disorders (PSSD) are under-reported and under-treated, despite their impact on recovery, quality of life, and post-stroke depression. Although polysomnography (PSG) is the gold standard for diagnosis, its limited availability leads to underdiagnosis.

**Aims:**

To assess the prevalence of PSSD based on patient- and caregiver-reported data, and to evaluate their concordance with findings from overnight PSG in post-stroke patients.

**Methods:**

In this cross-sectional study, adult patients (aged ≥18 years) with ischemic or hemorrhagic stroke (1 month to 1 year post-onset) were assessed. Sleep-related history was obtained from patients and caregivers. Sleep quality was evaluated using the Pittsburgh Sleep Quality Index (PSQI), risk of obstructive sleep apnea (OSA) was assessed with the STOP-BANG questionnaire, and depression and anxiety were evaluated with the Hamilton Depression Rating Scale (HAM-D) and Hamilton Anxiety Rating Scale (HAM-A), respectively. Stroke severity and outcomes were evaluated using the modified Rankin Scale (mRS) and Stroke-Specific Quality of Life Scale (SS-QoL). A subset underwent overnight PSG using a 14-channel SOMNOmedics system, analyzed per American Academy of Sleep Medicine (AASM) criteria. The Apnea-Hypopnea Index (AHI) was used to quantify sleep-disordered breathing.

**Results:**

Out of 103 enrolled patients, 41 (39.8%) underwent PSG. While only 23.3% (*n*=24) of patients and 11.7% (*n*=12) of caregivers independently reported sleep disturbances after stroke, specific questioning increased detection to 62%. PSG revealed obstructive sleep apnea (OSA) in 62% of those denying sleep issues and in 100% of those self-reporting problems. Periodic limb movement disorder in sleep (PLMS) was present in 34.5% of asymptomatic individuals. Higher STOP-BANG scores and longer stroke duration were seen in the PSG group. Wakefulness after sleep onset (WASO) >120 minutes was more common in patients with PSQI >5. AHI >5 was present in 65.8%, but not associated with any demographic, clinical, or questionnaire-based variables.

**Conclusion:**

There is poor correlation between self/caregiver-reported sleep problems and PSG-confirmed diagnoses in post-stroke patients. Proactive screening using structured questionnaires and PSG (or alternatives such as actigraphy) is essential in resource-limited settings to detect and treat sleep disorders that may impact recovery.

## Introduction

Post-stroke sleep disorders (PSSDs) are under-reported and under-treated. They have been shown to adversely affect patients’ quality of life and are associated with poor recovery and post-stroke depression (1). Global estimates suggest that 30 to 70% of stroke survivors experience at least one form of sleep disorder, depending on the population, stroke type, and diagnostic approach used (2, 3). A recent hospital-based study reported a prevalence of over 50% for PSSDs (4), underscoring its growing public health relevance in low- and middle-income countries, where both stroke incidence and long-term disability are on the rise.

The neurobiological basis of PSSDs is multifactorial. Stroke can disrupt sleep-regulating neural structures involved in the hypothalamus, thalamus, brainstem, and reticular activating system, leading to disruptions in circadian rhythm, arousal regulation, and respiratory control (5, 6).

While the majority of evidence on PSSDs focuses on sleep-disordered breathing (SDB) and obstructive sleep apnea (OSA) (2), there is a lack of data on other sleep disorders such as insomnia, restless legs syndrome (RLS), and periodic limb movements of sleep (PLMS). PSSDs may co-exist with post-stroke depression (7), which might contribute to and be associated with sleep disturbances. Many patients are unaware of their sleep problems, particularly in the presence of poor awareness, aphasia, or cognitive impairment. Moreover, healthcare providers may not routinely screen for sleep problems. Limited data are available comparing self-reported, caregiver-reported, and objective assessments of sleep disturbances in stroke survivors. Polysomnography (PSG) is the gold standard for diagnosing sleep disorders and is more sensitive than a questionnaire or clinical history in detecting conditions such as OSA or PLMS (8). However, its use in stroke patients is limited by reduced mobility, cognitive issues, and in-hospital logistical constraints. In many low-resource settings, PSG is neither feasible nor available, leading to underdiagnosis and undertreatment of PSSDs.

In this single-center, hospital-based, cross-sectional study, we aimed to determine the prevalence of PSSDs based on self-reports, questionnaire responses, and PSG findings. We further aimed to explore the concordance between subjective reports from patients and caregivers and objective PSG findings to evaluate the reliability of clinical assessments in detecting sleep disorders after stroke.

## Methods

### Study design and participants

**Figure 1:**
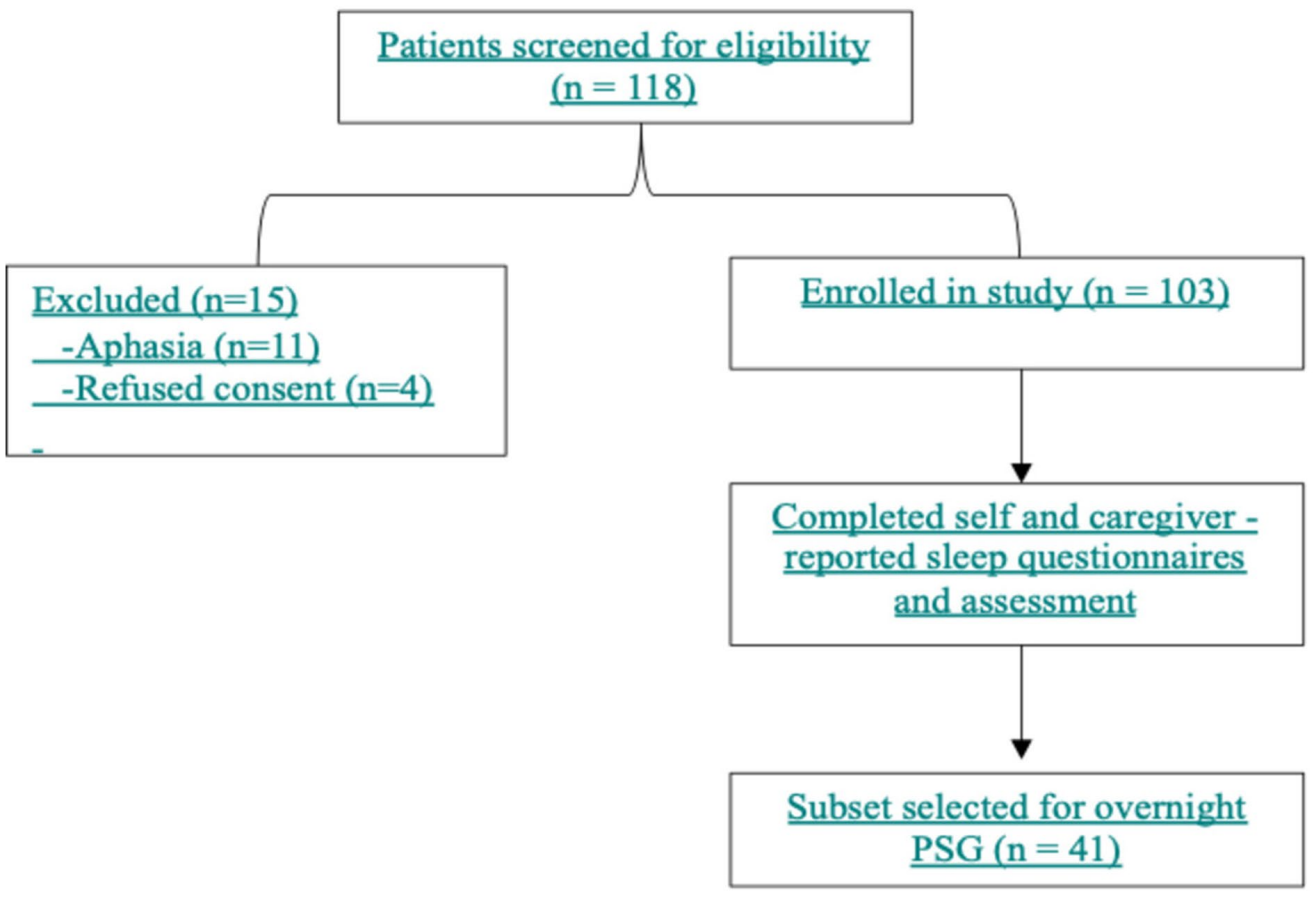
Study flow diagram.

This was a single-center, hospital-based, cross-sectional study conducted between January 2021 and June 2022. It was embedded within a larger study evaluating the prevalence and determinants of PSSDs (4) (Figure 1).

In the main study, all consecutive post-stroke patients (between 1 month and 1 year after stroke onset) and their caregivers were interviewed regarding various sleep problems. The study population included adult patients (≥18 years) with ischemic or hemorrhagic stroke who were between 1 month and less than 1 year post-event. Patients who were severely aphasic, dysarthric, tracheostomized, or unable to cooperate with the questionnaires were reassessed after 3 months to determine their eligibility. If still found ineligible, they were excluded from the study. Written informed consent was obtained from all patients. Ethical clearance for the study was granted by the institutional ethics committee (Reference no. IECPG- 557/21.10.2020, RT-11/25.11.2020).

### Assessment tools and data collection

The eligible and consenting patients underwent a detailed history and clinical examination. The patients and their primary caregivers initially completed a screening questionnaire with the following items: 1. Did you have sleep problems before the stroke? If yes, have they changed after the stroke? If no, do you have sleep problems after the stroke? 2. Have you ever discussed your sleep problems with a doctor? 3. Has your doctor ever enquired about any sleep problems? 4. Have you ever been evaluated for sleep problems? 5. For the caregiver: Does your patient have sleep problems? If yes, enumerate. If no, does he/she experience insomnia, daytime sleepiness, snoring, or pain in the limbs that disturbs sleep?

Quality of sleep in the patients was evaluated using the Pittsburgh Sleep Quality Index (PSQI) (9). It contains 19 questions related to the previous month’s sleeping habits. The PSQI is divided into seven components: sleep quality, latency, duration, efficiency, sleep disturbances, use of sleeping medication, and daytime dysfunction. The total score ranges from 0 to 21, and a score > 5 indicates poor sleep quality. Screening for obstructive sleep apnea (OSA) was performed using the STOP-BANG Questionnaire (10). Risk levels were classified as low (yes to 0–2 questions), intermediate (yes to 3–4 questions), and high (yes to 5–8 questions). This tool was selected as it is simple to administer and has demonstrated a high diagnostic accuracy of >0.8 (11). The following assessments were also performed: (A) the Montreal Cognitive Assessment (MoCA) (12) for cognitive screening; (B) the Hamilton Anxiety Rating Scale (HAM-A) (13) for post-stroke anxiety (PSA)(score range: 0–56, with < 17 indicating mild anxiety, 18–24 mild to moderate anxiety, 25–30 moderate to severe anxiety, and > 30 severe anxiety); (C) the Hamilton Depression Rating Scale (HAM-D) (14) for post-stroke depression (score interpretation: 0–7 normal, 8–13 mild depression, 14–18 moderate depression, 19–22 severe depression, and ≥ 23 very severe depression); (D) the modified Rankin Scale (mRS) (15) for motor disability; and (E) the Stroke Specific Quality of Life (SS-QOL) scale (16). The SS-QOL questionnaire assesses 12 domains: mobility, energy, upper extremity function, work and productivity, mood, self-care, social roles, family roles, vision, language, thinking, and personality. The total score ranges from 49 to 245, with higher scores indicating better functioning. The Trial of Org 10,172 in Acute Stroke Treatment (TOAST) (17) was used for stroke classification.

### Polysomnography subgroup

A subset of patients was selected for overnight polysomnography (PSG) based on the availability of laboratory slots and willingness to participate. PSG was not performed in all patients due to limited laboratory capacity, the COVID-19 pandemic, and logistical constraints. This may have introduced selection bias.

PSG was performed using a 14-channel SOMNOmedics sleep system (SOMNOmedics GmbH, Germany). PSG data were scored by a blinded assessor (MT) using the American Academy of Sleep Medicine (AASM) Manual for the Scoring of Sleep and Associated Events: Rules, Terminology and Technical Specifications, Version 2.6 (2020). The selection of patients for PSG was based on obtaining separate informed consent for the procedure.

### Sample size and power consideration

This study was exploratory and embedded within a larger cross-sectional study on PSSDs. Therefore, a formal sample size calculation was not performed for the PSG subgroup. A *post hoc* power analysis was not feasible due to the variability in PSG-detected outcomes and the limited number of patients who underwent PSG (*n* = 41). The findings should be interpreted as hypothesis-generating.

### Statistical analysis

Descriptive statistics were presented as proportions/percentages for categorical variables and as means with standard deviations for continuous variables. Normalcy of the data was tested using the Kolmogorov–Smirnov test. The self-reported questionnaires were compared with the PSG findings. Furthermore, the association between sleep quality (PSQI) and the PSG findings was analyzed. Finally, the association between demographic, clinical, and stroke characteristics and the PSG findings was also examined. For this comparison, the Apnoea–Hypopnea Index (AHI) was used as the dependent variable. Fisher’s exact test or chi-squared test was used for the comparison of proportions (Categorical variables). Continuous variables were analyzed using the Mann–Whitney U test or Student’s *t*-test (Independent group/Unpaired data) and Wilcoxon signed-rank test or paired *t*-test (for paired data) based on the normality of the data. While univariate analyses were performed, a multivariate logistic regression model was not applied due to the limited sample size and the risk of model overfitting. Statistical analyses were carried out using the statistical software STATA version 12.0.

## Results

During the study period (January 2021 to June 2022), 118 patients were screened for eligibility, of whom 15 were excluded (11 due to aphasia and four who refused participation). A total of 103 patients were enrolled in the study, of whom 41 (39.8%) underwent PSG. Upon administering the screening questionnaire regarding awareness of sleep disorders, only 23.3% (*n* = 24) of the patients and 11.7% (*n* = 12) of the caregivers independently reported sleep disturbances after stroke. This increased to 62% after asking specific questions (Table 1). The reported sleep problems included insomnia (difficulty falling/staying asleep), loud snoring, excessive daytime sleepiness, limb discomfort suggestive of RLS, and fragmented night sleep. Among the 41 patients who underwent PSG, OSA was detected in 62.1% (18/29) of those who denied having sleep problems—highlighting a significant proportion with subclinical disease—100% (12/12) of those who self-reported or whose caregivers reported sleep problems (Table 1). In addition, PLMS was present in 34.5% (10/29) of the patients who did not report any sleep disturbance, suggesting a high rate of undetected PLMS. It was present in 50–100% of the patients where sleep disturbances were reported by the patients and/or caregivers. The demographic characteristics of the patients who underwent PSG and the remaining study population were similar, except that the PSG group had higher STOP-BANG scores and a longer duration since stroke (see Supplementary Table S1). The PSG features were also similar between the patients with a PSQI score of >5 (73.4%) and those with normal PSQI scores. Only wakefulness after sleep onset (WASO) of >120 min was significantly more common in the group with a PSQI score >5 (Supplementary Table S2). An AHI > 5 was found in 65.8% of the patients. When the clinical and stroke characteristics, as well as other questionnaire scores, were compared between the patients with normal and high AHI (>5), no significant differences were observed (Supplementary Table S3). There were no missing values in the primary outcome data (PSG, questionnaires).

**Table 1:** Assessment of awareness of sleep disorders and association with PSG findings.

Question	Number [*n* = 103 (%)]
For the patient	
1. Did you have sleep problems before the stroke?	16 (15.5)
2. If yes, have the sleep problems changed after stroke (yes)?	16 (15.5)
3. Do you have sleep problems after stroke?	24 (23.3)
4. Have you discussed your sleep problems with a doctor?	0 (0)
5. Has your doctor ever enquired about any sleep problems?	0 (0)
For the caregiver	
1. Does your patient have any sleep problems?	12 (11.7)
2. Patient reported sleep problem but denied by caregiver	6 (5.8)
3. Sleep problem denied by patient but reported by caregiver	12 (11.7)
4. Sleep problem reported by both patient and caregiver	10 (9.7)
5. Both patient and caregiver do not report any sleep problem	75 (72.8)
Sleep problems reported by patient/caregiver after asking specifically	
1. Overall	62 (60.2)^a^
2. Insomnia	21(20.4)
3. Snoring	50 (48.5)
4. Excessive daytime sleepiness	3 (2.9)
5. Abnormal leg movements in sleep	0 (0)

Any sleep problem?		Number	Polysomnographic findings			
Patients	Caregiver	(*n* = 41)	OSA	PLMS	Disrupted sleep	Prolonged latency
			*N* = 27 (65.9%)	*N* = 17 (41.5%)	*N* = 14 (34%)	*N* = 15 (36.6%)
Yes	No	2	2 (100%)	1 (50%)	2 (100%)	1 (50%)
No	Yes	6	5 (83.3%)	3 (50%)	2 (33.3%)	2 (33.3%)
Yes	Yes	3	2 (67.7%)	3 (100%)	1 (33.3%)	1 (33.3%)
No	No	29	18 (62%)	10 (34.5%)	9 (31%)	11 (37.9%)

a Some patients/caregivers reported more than one sleep problem.

Shows the number and percentage of post-stroke patients (*n* = 103) and caregivers reporting sleep problems before and after structured questioning, and cross-tabulation with PSG findings in the PSG subgroup (*n* = 41). OSA, obstructive sleep apnea; PLMS, periodic limb movement in sleep. Statistical notes: percentages based on total cohort (*n* = 103) or PSG subgroup (*n* = 41) as indicated. Some respondents reported multiple problems.

## Discussion

Post-stroke sleep disturbance affects patients’ quality of life. This study found that even among patients with self- or caregiver-reported sleep disturbances, PSG detected a higher prevalence of various sleep disturbances. There was a disparity between the prevalence of sleep problems reported by the patients and caregivers and those confirmed through the questionnaires. This disparity further widened when sleep disorders were confirmed by PSG. Another interesting finding was that, when specifically examining the AHI, there was no association between AHI > 5 and any specific clinical, stroke-related, or questionnaire-based findings. Several population- and hospital-based studies have shown that poor sleep quality is associated with worse cognitive function (18), reduced quality of life (18), depression, and impaired motor recovery (19). Our findings of low spontaneous reporting but significantly higher detection through structured questioning align with previous observations that sleep disorders are frequently underreported after stroke due to factors such as cognitive impairment, aphasia, low patient awareness of sleep symptoms, and the lack of routine screening during follow-up care (20).

OSA is the most extensively studied post-stroke sleep disorder. Population-based and hospital-based studies report that 50–60% of stroke survivors have sleep-disordered breathing (SDB), with a substantial proportion having moderate-to-severe OSA (2, 4). In our PSG subgroup, 65.8% of the patients had an Apnoea–Hypopnea Index (AHI) > 5, consistent with prior reports. Stroke can both unmask pre-existing OSA and induce or exacerbate it via mechanisms such as impaired upper airway muscle tone, altered ventilatory control (especially in brainstem lesions), and changes in sleep architecture and body position following the stroke, such as increased time spent sleeping in the supine position (21). Untreated OSA after stroke is associated with an increased risk of recurrent events, poorer functional recovery, cognitive impairment, and mood disturbances (22). Our finding that many patients who denied sleep complaints were nonetheless diagnosed with OSA on PSG underscores the silent nature of SDB and highlights the importance of routine screening.

Meta-analyses estimate a pooled prevalence of post-stroke insomnia at approximately 38% (95% CI 30.1–46.5%) among stroke survivors—significantly higher compared to the general population (23). Insomnia after stroke may result from lesion-related disruption of sleep-regulatory networks (e.g., thalamus, hypothalamus), as well as neuroinflammation, pain, anxiety, depression, and hospital environment factors in the acute phase (5, 6). In our cohort, structured questioning revealed insomnia symptoms (difficulty initiating/maintaining sleep) in a subset of patients; however, formal PSG criteria for insomnia (e.g., sleep latency, fragmentation) may be missed without objective recording.

The prevalence of PLMS post-stroke has been reported to range between 27 and 48% in the subacute and chronic phases, with some studies showing even higher rates in the acute phase (e.g., up to 76% in acute stroke cohorts) (24–26). Mechanisms underlying PLMS after stroke may include white matter and basal ganglia lesions disrupting spinal and supraspinal pathways, altered dopaminergic neurotransmission, and coexisting SDB (20). RLS can also emerge or worsen post-stroke, particularly with lesions in regions such as the caudate, thalamus, or brainstem (27). In our study, we observed PLMS even among the patients who denied sleep complaints, suggesting under-recognition. Given emerging evidence that PLMS and co-existing SDB may negatively influence recovery and quality of life, early detection is important.

Therefore, the under-detection of sleep disorders may be a significant contributor to poor quality of life in post-stroke patients. Some sleep disorders such as PLMS are reported even less. There is a research gap regarding the relationship between PLMS and stroke (2). While OSA is the most frequently studied PSSDs, with specific treatment guidelines (2), insomnia and PLMS also need to be adequately diagnosed and treated. Early identification and treatment of PSSDs can favorably influence neurorehabilitation, cognitive recovery, mood, and reduce recurrent stroke risk. Therefore, it is crucial to integrate sleep screening into standard stroke follow-up protocols.

Full in-lab PSG is often impractical post-stroke due to mobility, cognitive, and logistic constraints. Portable/home-based PSG or polygraphy may offer feasible alternatives but require validation in this population. Meanwhile, actigraphy provides a low-cost method to monitor sleep patterns, although it cannot diagnose OSA/PLMS. A tiered approach—screening with questionnaires and actigraphy, followed by targeted PSG—can be pragmatic in resource-limited settings. It is crucial to educate caregivers to help them recognize sleep disorder signs (e.g., apnea, restless movements), maintain sleep diaries or use actigraphy, and support adherence to treatments (e.g., CPAP, sleep hygiene). Empowering them raises awareness of the importance of sleep in recovery and prompts timely evaluation and intervention.

Our study has several limitations. PSG could not be performed in all patients, which may have introduced selection bias, as patients with sleep problems might have been more likely to consent. The sample size was also small, especially for comparisons within the subgroup of patients who underwent PSG. In addition, there was no follow-up. Therefore, we cannot determine whether treatment of the diagnosed sleep problems had any benefit. The PSG findings should be interpreted with caution due to the potential first-night effect, which may lead to overdiagnosis of insomnia. Studies using home-based actigraphy in post-stroke patients have detected both overestimation and underestimation of self-reported sleep duration (28). The strengths of the study include the novelty of its findings, highlighting that proactive assessment of PSSDs should be conducted using questionnaires and, preferably, PSG. Another strength is the inclusion of family caregivers in the assessment, which improved the yield by approximately 10%.

In conclusion, the study highlights the under-reported and under-diagnosed burden of PSSDs. In an ideal setting, most post-stroke patients with clinical suspicion of PSSDs should undergo PSG. However, given limited resources and the challenges of in-hospital PSG, it is advisable to integrate structured sleep screening tools (e.g., STOP-BANG Questionnaire, PSQI, RLS criteria) along with caregiver input into routine stroke follow-up. Initial assessments can be conducted using questionnaires and actigraphy, with targeted PSG reserved for high-risk cases. Educating caregivers to recognize sleep symptoms may also help in early intervention. Advocacy for policies and resources to improve access to sleep diagnostics in resource-limited settings is essential to enhance recovery and reduce stroke recurrence. There is already evidence demonstrating that treatment of sleep-disordered breathing (SDB) reduces stroke recurrence (29, 30). Further efforts to detect and treat other sleep disorders are needed to improve stroke outcomes.

## Data Availability

The original contributions presented in the study are included in the article/Supplementary material, further inquiries can be directed to the corresponding author.

## References

[1] Sonmez I Karasel S. Poor sleep quality I related to impaired functional status following stroke. J Stroke Cerebrovasc Dis. (2019) 28:104349. doi: 10.1016/j.jstrokecerebrovasdis.2019.104349, PMID: 31492629

[2] Bassetti CLA Randerath W Vignatelli L Ferini-Strambi L Brill AK Bonsignore MR. EAN/ERS/ESO/ESRS statement on the impact of sleep disorders on risk and outcome of stroke. Eur Respir J. (2020) 55:1901104. doi: 10.1183/13993003.01104-2019, PMID: 32317355

[3] Johnson KG Johnson DC. Frequency of sleep apnea in stroke and TIA patients: a meta-analysis. J Clin Sleep Med. (2010) 6:131. doi: 10.5664/jcsm.27760 20411688 PMC2854698

[4] Tayade K Vibha D Singh RK Pandit AK Ramanujam B Das A. Prevalence and determinants of post-stroke sleep disorders: a cross-sectional hospital-based study. Sleep Breath. (2023) 27:2429–33. doi: 10.1007/S11325-023-02850-Z, PMID: 37183196

[5] Hermann DM Bassetti CL. Sleep-related breathing and sleep-wake disturbances in ischemic stroke. Neurology. (2009) 73:1313–22. doi: 10.1212/WNL.0b013e3181bd137c, PMID: 19841384

[6] Bassetti C Aldrich MS. Sleep apnea in acute cerebrovascular diseases: final report on 128 patients. Sleep. (1999) 22:217–23. doi: 10.1093/SLEEP/22.2.217, PMID: 10201066

[7] Nguyen TTP Nguyen TX Nguyen TC Nguyen HTT Nguyen TN Nguyen TTH. Post-stroke depression in Vietnamese patients is associated with decreased sleep quality and increased fatigue: a one-institution cross-sectional analysis. Sleep Breath. (2023) 27:1629–37. doi: 10.1007/S11325-022-02745-5, PMID: 36434377 PMC9702659

[8] Sforza E Roche F. Sleep apnea syndrome and cognition. Front Neurol. (2012) 3:87. doi: 10.3389/FNEUR.2012.00087 22661967 PMC3361858

[9] Smyth C. The Pittsburgh sleep quality index (PSQI). J Gerontol Nurs. (1999) 25:10–1. doi: 10.3928/0098-9134-19991201-10, PMID: 10711108

[10] Chung F Abdullah HR Liao P. STOP-bang questionnaire: a practical approach to screen for obstructive sleep apnea. Chest. (2016) 149:631–8. doi: 10.1378/CHEST.15-0903, PMID: 26378880

[11] Pivetta B Chen L Nagappa M Saripella A Waseem R Englesakis M. Use and performance of the STOP-bang questionnaire for obstructive sleep apnea screening across geographic regions: a systematic review and Meta-analysis. JAMA Netw Open. (2021) 4:e211009–9. doi: 10.1001/JAMANETWORKOPEN.2021.1009, PMID: 33683333 PMC7941199

[12] Nasreddine ZS Phillips NA Bédirian V Charbonneau S Whitehead V Collin I. The Montreal cognitive assessment, MoCA: a brief screening tool for mild cognitive impairment. J Am Geriatr Soc. (2005) 53:695–9. doi: 10.1111/J.1532-5415.2005.53221.X, PMID: 15817019

[13] Hamilton M. The assessment of anxiety states by rating. Br J Med Psychol. (1959) 32:50–5. doi: 10.1111/J.2044-8341.1959.TB00467.X, PMID: 13638508

[14] Hamilton M. A rating scale for depression. J Neurol Neurosurg Psychiatry. (1960) 23:56–62. doi: 10.1136/JNNP.23.1.56, PMID: 14399272 PMC495331

[15] Hacke W Kaste M Fieschi C Toni D Lesaffre E Von Kummer R. Intravenous thrombolysis with recombinant tissue plasminogen activator for acute hemispheric stroke: the European cooperative acute stroke study (ECASS). JAMA. (1995) 274:1017–25. doi: 10.1001/JAMA.1995.03530130023023 7563451

[16] Williams LS Weinberger M Harris LE Clark DO Biller J. Development of a stroke-specific quality of life scale. Stroke. (1999) 30:1362–9. doi: 10.1161/01.STR.30.7.1362, PMID: 10390308

[17] Adams HP Davis PH Leira EC Chang KC Bendixen BH Clarke WR. Baseline NIH stroke scale score strongly predicts outcome after stroke: a report of the trial of org 10172 in acute stroke treatment (TOAST). Neurology. (1999) 53:126–31. doi: 10.1212/WNL.53.1.126, PMID: 10408548

[18] Fan X-W Yang Y Wang S Zhang YJ Wang AX Liao XL. Impact of persistent poor sleep quality on post-stroke anxiety and depression: a National Prospective Clinical Registry Study. Nat Sci Sleep. (2022) 14:1125–35. doi: 10.2147/nss.s357536, PMID: 35721879 PMC9205438

[19] Fleming MK Smejka T Henderson Slater D Chiu EG Demeyere N Johansen-Berg H. Self-reported and objective sleep measures in stroke survivors with incomplete motor recovery at the chronic stage. Neurorehabil Neural Repair. (2021) 35:851–60. doi: 10.1177/15459683211029889, PMID: 34196598 PMC8442123

[20] Hasan F Gordon C Wu D Huang HC Yuliana LT Susatia B. Dynamic prevalence of sleep disorders following stroke or transient ischemic attack: systematic review and meta-analysis. Stroke. (2021) 52:655–63. doi: 10.1161/STROKEAHA.120.029847, PMID: 33406871

[21] Lv R Liu X Zhang Y Dong N Wang X He Y. Pathophysiological mechanisms and therapeutic approaches in obstructive sleep apnea syndrome. Signal Transduct Target Ther. (2023) 8:218–46. doi: 10.1038/s41392-023-01496-3, PMID: 37230968 PMC10211313

[22] Baillieul S Dekkers M Brill AK Schmidt MH Detante O Pépin J-L. Sleep apnoea and ischaemic stroke: current knowledge and future directions. Lancet Neurol. (2022) 21:78–88. doi: 10.1016/S1474-4422(21)00321-5, PMID: 34942140

[23] Baylan S Griffiths S Grant N Broomfield NM Evans JJ Gardani M. Incidence and prevalence of post-stroke insomnia: a systematic review and meta-analysis. Sleep Med Rev. (2020) 49:101222. doi: 10.1016/J.SMRV.2019.101222, PMID: 31739180

[24] Bassetti CL Milanova M Gugger M. Sleep-disordered breathing and acute ischemic stroke: diagnosis, risk factors, treatment, evolution, and long-term clinical outcome. Stroke. (2006) 37:967–72. doi: 10.1161/01.STR.0000208215.49243.c3, PMID: 16543515

[25] Bassetti CL Aldrich MS. Sleep electroencephalogram changes in acute hemispheric stroke. Sleep Med. (2001) 2:185–94. doi: 10.1016/S1389-9457(00)00071-X, PMID: 11311681

[26] Plomaritis P Theodorou A Michalaki V Stefanou M-I Palaiodimou L Papagiannopoulou G. Periodic limb movements during sleep in acute stroke: prevalence, severity and impact on post-stroke recovery. J Clin Med. (2023) 12:5881. doi: 10.3390/JCM12185881, PMID: 37762823 PMC10531709

[27] Tinh DX Hung DV Thuan DD Duc DP Duc DM Cuong ND. Stroke-related restless leg syndrome in hemorrhagic and ischemic stroke patients. SAGE Open Med. (2025) 13:20503121251336900. doi: 10.1177/20503121251336900 40309317 PMC12041691

[28] Jackson CL Ward JB Johnson DA Sims M Wilson J Redline S. Concordance between self-reported and actigraphy-assessed sleep duration among African-American adults: findings from the Jackson heart sleep study. Sleep. (2020) 43:zsz246. doi: 10.1093/SLEEP/ZSZ246, PMID: 31616945 PMC7066489

[29] Gupta A Shukla G Afsar M Poornima S Pandey RM Goyal V. Role of positive airway pressure therapy for obstructive sleep apnea in patients with stroke: a randomized controlled trial. J Clin Sleep Med. (2018) 14:511–21. doi: 10.5664/JCSM.7034, PMID: 29609704 PMC5886428

[30] McEvoy RD Antic NA Heeley E Luo Y Ou Q Zhang X. CPAP for prevention of cardiovascular events in obstructive sleep apnea. N Engl J Med. (2016) 375:919–31. doi: 10.1056/NEJMOA1606599 27571048

