# Supplementary material for: Polysomnographic correlates of self-and caregiver-reported sleep problems in post-stroke patients
Source: Front Neurol. 2025 Jul 16;16:1587378. doi: 10.3389/fneur.2025.1587378 (PMC12307309; doi:10.3389/fneur.2025.1587378)
Supplement: Supplementary file 1 [file Data_Sheet_1.docx]

# Table S1:

| **Variable** | **N= 103** | **PSG done.**  **(n=41)** | **Not done (n=62)** | **P value** |
| --- | --- | --- | --- | --- |
| **Demographic features** | |  |  |  |
| Age, years (SD) | 50.7 (11.7) | 51.4 (8.4) | 50.3(13.5) | 0.63 |
| Upto 60 years, n (%) | 85 (82) | 37 (90.2) | 48 (77.4) | 0.07 |
| Sex, male, n (%) | 76 (73.8) | 33 (80.5) | 43 (69.4) | 0.21 |
| Residential area, urban, n (%) | 83 (80.6) | 36 (87.8) | 47 (75.8) | 0.13 |
| Stroke type, ischemic, n (%) | 88 (85.4) | 35 (85.4) | 53 (85.5) | 0.99 |
| Hemisphere involved, n (%) |  |  |  |  |
| Right | 59 (57.3) | 29 (70.7) | 30 (48.4) | **0.05** |
| Left | 40 (38.8) | 10 (24.4) | 30 (48.4) |  |
| Bilateral | 4 (3.9) | 2 (4.9) | 2 (3.2) |  |
| **Risk factors, n (%)** | |  |  |  |
| BMI>24 | 76 (73.8) | 32 (78.0) | 46 (74.2) | 0.66 |
| Diabetes mellitus | 26 (25.2) | 11 (26.8) | 15 (24.2) | 0.76 |
| Hypertension | 57 (55.3) | 23 (56.1) | 33 (53.2) | 0.78 |
| Prior stroke | 14 (13.6) | 4 (9.8) | 10 (16.1) | 0.36 |
| CAD | 6 (5.8) | 2 (4.9) | 4 (6.5) | 0.55 |
| Atrial fibrillation | 4 (3.9) | 0 | 4 (6.5) | 0.13 |
| Smoking | 35 (34.0) | 21 (51.2) | 25 (40.3) | 0.28 |
| Alcohol use | 36 (35) | 17 (41.5) | 19 (30.6) | 0.26 |
| **Sleep related scales, median (IQR)** | | | | |
| PSQI | 8.0(5.0;11.0) | 8.0 (4.5;11.5) | 8.0 (4.8;11.0) | 0.53 |
| PSQI>5 | 74 (71.8) | 30 (73.2) | 44 (71.0) | 0.80 |
| STOP-BANG | 4.0 (3.0;5.0) | 4.0 (3.0;5.5) | 3.0 (2.0;5.5) | **0.01** |
| High risk OSA (5-8) | 34 (33.0) | 18 (43.9) | 16 (25.8) | 0.05 |
| Duration post-stroke, months | 2.7 (1.4;6.7) | 4.2 (1.5;9.0) | 2.5 (1.4;5.2) | **0.04** |
| **Co-variates, median (IQR)** | | | | |
| SSQoL | 208.0 (179.0; 235.0) | 222.0 (182.0;236.0) | 207.0 (173.7;234.3) | 0.58 |
| MoCA | 23.0(18.0;27.0) | 24.0(19.5;27.0) | 22.0(17.0;25.5) | 0.06 |
| HAM-A | 6.0 (3.0; 9.0) | 6.0 (2.0; 10.0) | 6.0 (1.0; 9.0) | 0.25 |
| HAM-D | 4.0 (1.0; 6.0) | 4.0 (1.0; 7.0) | 3.5 (0.0; 6.0) | 0.61 |
| **Stroke severity scales, median (IQR)** | | | | |
| NIHSS (ictus) | 8.0 (5.0;14.0) | 10.0 (5.0;14.0) | 8.0 (5.0;14) | 0.43 |
| NIHSS (enrollment) | 2.0 (1.0;5.0) | 3.0 (1.0;5.0) | 2.0 (1.0;5.0) | 0.59 |
| mRS (ictus) | 4.0 (2.0;5.0) | 4.0 (3.0;5.0) | 4.0 (2.0;5.0) | 0.35 |
| mRS (enrollment) | 1.0 (1.0;2.0) | 1.0 (1.0;2.0) | 1.0 (1.0;2.3) | 0.64 |

**Table S1. Comparison of clinical and demographic features in patients who underwent PSG versus those who did not**

Compares baseline characteristics (demographics, vascular risk factors, sleep-related scales, functional and cognitive scores) between the PSG subgroup (n=41) and non-PSG group (n=62). Continuous variables reported as mean±SD or median (IQR) as appropriate; categorical variables as n (%). Statistical tests: independent t-test or Mann–Whitney U test for continuous variables; chi-square or Fisher’s exact test for categorical variables. Abbreviations: PSG, polysomnography; PSQI, Pittsburgh Sleep Quality Index; SS-QoL, Stroke-Specific Quality of Life; MoCA, Montreal Cognitive Assessment; HAM-A, Hamilton Anxiety Rating Scale; HAM-D, Hamilton Depression Rating Scale; NIHSS, National Institutes of Health Stroke Scale; mRS, modified Rankin Scale; OSA, obstructive sleep apnea; BMI, body mass index.

# Table S2:

| **PSG Parameter** | | **Overall**  **(N = 41)** | **PSQI** $\boldsymbol{\leq}$**5**  **(N = 11)** | **PSQI > 5**  **(N = 30)** | **P Value** |
| --- | --- | --- | --- | --- | --- |
| Sleep Efficiency | >80% | 23 | 5 (45.5) | 18 (60.0) | 0.41 |
| Sleep Latency | >30 min | 15 | 4 (36.4) | 11(36.7) | 0.99 |
| AHI $(Median\pm$ IQR) | | 14.3 (3.8;24.0) | 16.8 (3.8; 31.4) | 12.4 (3.8;23.4) | 0.48 |
| AHI | >5 | 27 (65.8) | 7 (63.6) | 20 (66.7) | 0.86 |
| Arousal Index | >25 | 5 (12.2) | 2 (18.2) | 3 (10.0) | 0.48 |
| PLM Index | >15 | 16 (39.0) | 4 (36.4) | 12 (40.0) | 0.83 |
| WASO | >120 min | 14 (34.1) | 1 (9.1) | 13 (43.3) | 0.04 |
| WASO (% of sleep) | >20% | 25 (61.0) | 5 (45.5) | 20 (66.7) | 0.22 |
| Lowest Saturation (Mean$\pm$ SD) | | 84.3 ± 6.6 | 86.1 ± 5.2 | 83.6 ±6.9 | 0.29 |
| Desaturation Index  Mean$\pm$ SD | | 15.8 ± 13.3 | 16.8 ±14.9 | 15.4 ±12.9 | 0.77 |

**Table S2. Association between sleep quality (PSQI) and polysomnographic features in the PSG subgroup**

Displays PSG parameters in patients stratified by PSQI score (≤5 vs >5) among those who underwent PSG (n=41). Parameters include sleep efficiency, sleep latency, AHI (median, IQR; proportion with AHI>5), arousal index, PLM index, WASO duration and percentage, oxygen saturation metrics. Data shown as n (%) for categorical thresholds or mean±SD/median (IQR) for continuous measures. Statistical tests: chi-square or Fisher’s exact for categorical; t-test or Mann–Whitney U for continuous. Abbreviations: PSQI, Pittsburgh Sleep Quality Index; AHI, Apnea–Hypopnea Index; PLM, periodic limb movement; WASO, wakefulness after sleep onset.

# Table S3:

| **Characteristic** | | **Overall**  **(N = 41)** | **AHI** $\boldsymbol{\leq}$**5**  **(N = 14)** | **AHI > 5**  **(N = 27)** | **p Value** |
| --- | --- | --- | --- | --- | --- |
| Age (mean$\pm SD$) | | 51.37 (8.43) | 48.21 (11.23) | 53 (6.17) | 0.08 |
| Males | | 27 | 12 (77.8) | 21 (85.7) | 0.54 |
| BMI (mean$\pm$ SD) | | 26.67 (3.89) | 25.38 (3.62) | 27.33 (3.91) | 0.13 |
| Diabetes Mellitus | | 11 | 4 (28.6) | 7 (25.9) | 0.86 |
| Hypertension | | 23 | 6 (42.9) | 17 (63.0) | 0.22 |
| Prior Stroke | | 4 | 2 | 2 | 0.48 |
| Coronary artery disease | | 2 | 1 (7.1) | 1 (3.7) | 0.63 |
| Atrial fibrillation | | 0 | - | - | - |
| Smoking | | 21 (51.2) | 7 (50.0) | 14 (51.9) | 0.91 |
| Alcohol use | | 17 | 7 (50.0) | 10 (37.0) | 0.42 |
| **Stroke Characteristic** | | | | | |
| Type | Ischemic | 35 | 10 | 25 | 0.07 |
| Arterial territory | | | | | |
|  | ACA | 1 | 0 | 1 | 0.52 |
|  | MCA | 22 | 6 | 16 | 0.83 |
|  | PCA | 8 | 2 | 6 | 0.8 |
|  | Basilar | 4 | 2 | 2 | 0.31 |
|  | Vertebral | 2 | 0 | 2 | 0.36 |
|  | SCA | 1 | 0 | 1 | 0.52 |
| TOAST | LAA | 16 | 3 | 13 | 0.08 |
|  | SVD | 7 | 1 | 6 |  |
|  | CE | 1 | 1 | 0 |  |
|  | Undetermined | 10 | 4 | 6 |  |
|  | Other determined | 1 | 1 | 0 |  |
| **Co-variates** | | | | | |
| HAM-A (mean$\pm$ SD) | | 7.09 (5.64) | 5.42 (4.14) | 7.96 (6.17) | 0.18 |
| HAM-D (mean$\pm$ SD) | | 4.07 (3.57) | 3.71 (3.58) | 4.25 (3.62) | 0.65 |
| STOP-BANG (mean$\pm$ SD) | | 4.34 (1.66) | 4.07 (1.59) | 4.48 (1.71) | 0.46 |
| MOCA (mean$\pm$ SD) | | 23.07 (4.56) | 23.71 (3.93) | 22.74 (4.89) | 0.52 |
| HAM-A | <17 | 39 | 14 | 25 | 0.30 |
|  | 18-24 | 2 | 0 | 2 |  |
| HAM-D | <7 | 34 | 11 | 23 | 0.54 |
|  | 8-13 | 6 | 3 | 3 |  |
|  | 14-18 | 1 | 0 | 1 |  |
| STOP-BANG | 0-2 | 4 | 1 | 3 | 0.60 |
|  | 3-4 | 19 | 8 | 11 |  |
|  | >5 | 18 | 5 | 13 |  |
| MoCA | ≤26 | 25 | 7 | 18 | 0.30 |
|  | >26 | 16 | 7 | 9 |  |
| SSQoL | Total | 206.2 (35.7) | 212.4 (33.6) | 203.0(37.0) | 0.43 |
|  | Mobility | 25.3 (6.7) | 27.35 (4.65) | 24.18 (7.44) | 0.15 |
|  | Energy | 11.9 (3.1) | 12.4 (3.3) | 11.6 (3.1) | 0.45 |
|  | UL function | 21.3 (5.3) | 22.4 (4.9) | 20.7 (5.5) | 0.35 |
|  | Work and Productivity | 11.7 (3.2) | 11.7 (3.2) | 11.7 (3.3) | 0.97 |
|  | Mood | 20.9 (4.0) | 21.1 (3.4) | 20.8 (4.4) | 0.87 |
|  | Self-Care | 21.4 (5.2) | 23.1 (3.4) | 20.6 (5.8) | 0.16 |
|  | Social roles | 21.4 (5.0) | 22.2 (3.9) | 21.0 (5.6) | 0.49 |
|  | Family Roles | 12.3 (3.2) | 12.9 (3.1) | 12.0 (3.2) | 0.38 |
|  | Vision | 13.9 (2.1) | 13.7 (2.5) | 14.1 (1.8) | 0.60 |
|  | Language | 22.7 (4.4) | 23.5 (3.6) | 22.2 (4.9) | 0.41 |
|  | Thinking | 10.8 (3.1) | 10.6 (4.0) | 11.0 (2.6) | 0.71 |
|  | Personality | 11.8 (3.1) | 11.5 (3.3) | 12.0 (3.0) | 0.63 |

**Table S3. Association between clinical characteristics and AHI category in PSG subgroup**

Compares demographic, vascular risk factors, stroke characteristics, and scale scores between patients with AHI ≤5 (n=14) and AHI >5 (n=27) among those who underwent PSG. Continuous variables reported as mean±SD; categorical as n (%). Statistical tests: independent t-test or Mann–Whitney U for continuous; chi-square or Fisher’s exact for categorical comparisons. Abbreviations: AHI, Apnea–Hypopnea Index; BMI, body mass index; HAM-A, Hamilton Anxiety Rating Scale; HAM-D, Hamilton Depression Rating Scale; MoCA, Montreal Cognitive Assessment; SS-QoL, Stroke-Specific Quality of Life; TOAST, Trial of Org 10172 in Acute Stroke Treatment classification; ACA, anterior cerebral artery; MCA, middle cerebral artery; PCA, posterior cerebral artery; SCA, superior cerebellar artery; LAA, large artery atherosclerosis; SVD, small vessel disease; CE, cardioembolism.
